# Somatic mutation that affects transcription factor binding upstream of *CD55* in the temporal cortex of a late-onset Alzheimer disease patient

**DOI:** 10.1093/hmg/ddz085

**Published:** 2019-04-24

**Authors:** Hafdis T Helgadottir, Pär Lundin, Emelie Wallén Arzt, Anna-Karin Lindström, Caroline Graff, Maria Eriksson

**Affiliations:** 1 Department of Biosciences and Nutrition, Center for Innovative Medicine, Karolinska Institutet, Huddinge, Sweden; 2 Science for Life Laboratory, Department of Biochemistry and Biophysics, Stockholm University, Stockholm, Sweden; 3 Department of Neurobiology, Care Sciences and Society, Center for Alzheimer Research, Division for Neurogeriatrics, Karolinska Institutet, Solna, Sweden; 4 Unit for Hereditary Dementias, Theme Aging, Karolinska University Hospital, Solna, Sweden

## Abstract

Alzheimer’s disease (AD) is the most common neurodegenerative disease worldwide. Familial cases suggest genetic components; however, monogenetic causes are few, and the vast majority of incidences have unknown cause. Sequencing efforts have focused on germline mutations, but improved technology has opened up for studies on somatic mutations in affected brain tissue samples. Here we use ultra-deep sequencing on brain and blood from early-onset AD (EOAD) and late-onset AD (LOAD) patients and non-AD individuals (*n* = 16). In total, 2.86 Mb of genomic regions, previously associated with AD, were targeted included 28 genes and upstream and downstream regulatory regions. Tailored downstream bioinformatics filtering identified 11 somatic single nucleotide variants in the temporal cortex in AD patients and none in the controls. One variant was validated to be present at 0.4% allele frequency in temporal cortex of a LOAD patient. This variant was predicted to affect transcription factor binding sites upstream of the *CD55* gene, contributing to AD pathogenesis by affecting the complement system. Our results suggest that future studies targeting larger portions of the genome for somatic mutation analysis are important to obtain an increased understanding for the molecular basis of both EOAD and LOAD.

## Introduction

Alzheimer’s disease (AD; OMIM 104300) is a neurodegenerative disorder mainly affecting elderly people and is the main cause for late-onset dementia. Cognitive functions are affected, causing memory impairment and personality changes. The disease is classified into early-onset AD (EOAD; onset before age 65 years) and late-onset AD (LOAD). While the *APOE* ɛ4 allele is the major genetic attributable risk factor for AD, mutations in the genes *APP* (amyloid precursor protein), *PSEN1* (presenilin 1) and *PSEN2* (presenilin 2) are known causes for autosomal dominant EOAD. Genome-wide association studies have reported several variants linked to AD (1), and mosaic loss of chromosome Y in the blood has been associated with the disease (2). However, most AD cases are sporadic with unknown causes.

**Figure 1:**
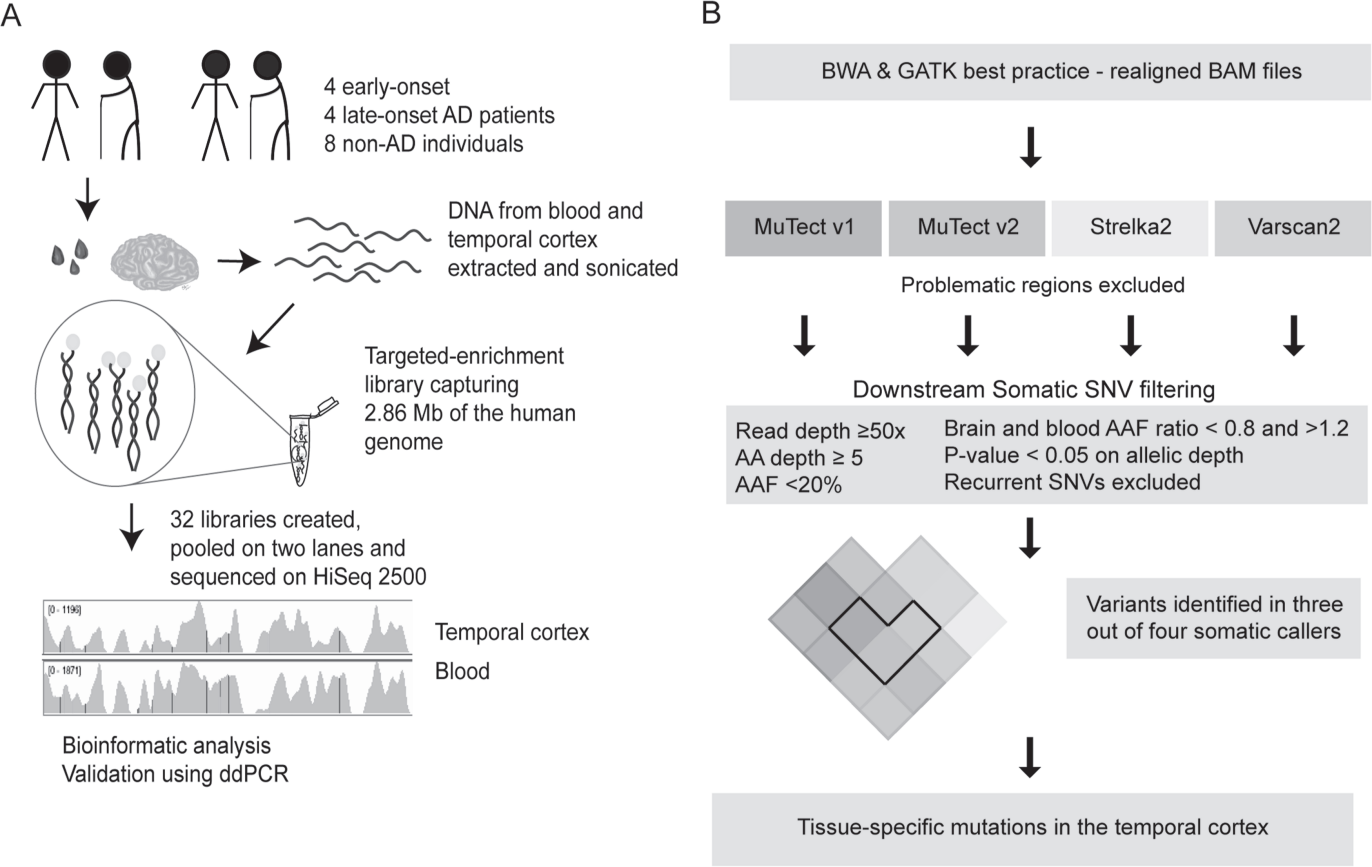
Workflow and bioinformatics overview. (**A**) Blood and brain (temporal cortex) samples were obtained from EOAD patients (*n* = 4), LOAD patients (*n* = 4) and age- and gender-matched non-AD individuals (*n* = 8). The DNA was extracted and sonicated before library preparation. During the library preparation, index primers were added to the DNA, and every sample was captured on an individual array containing 2.86 Mb of the human genome. The 32 libraries were mixed in 2 pools of 16 samples each and sequenced on HiSeq 2500, to be followed with bioinformatics downstream filtering to reveal brain-specific SNVs and validation by ddPCR. (**B**) Raw fastq files were aligned using BWA and processed using GATK-best practice that resulted in realigned BAM files. Variants were called using four somatic mutation callers, and downstream filtering was applied to identify tissue-specific somatic SNVs in the brain.

With advanced technology and bioinformatic analysis, somatic mutations in the brain have been identified (3–9), where each neuron is believed to have up to 1500 single nucleotide variants (SNVs) (4,5). Single-cell DNA sequencing has improved the ability to identify tissue-specific mutations (4,5). However, this method still faces many challenges (10). Compared with deep sequencing of unamplified bulk DNA, single-cell sequencing may introduce errors at the sequencing step during DNA amplification and its generally low genomic coverage can cause biases when identifying somatic mutations. Sequencing on AD-related genes (6–9) has shown that somatic mutations occur in the brain of AD patients, although it is unclear if the variants are pathogenic. Despite these findings, not all studies have successfully identified brain-specific mutations in bulk DNA (11,12). In order to achieve the required complexity and depth to detect rare somatic tissue-specific mutations in bulk tissue samples, comprehensive analysis and strict downstream filtering of ultra-deep sequencing data (>100× coverage) from high DNA input are needed. In this study, we used ultra-deep sequencing of DNA extracted from both the temporal cortex of the brain and blood to identify tissue-specific mosaic mutations in brain of AD patients and age-matched non-AD individuals. Using high DNA input, we created targeted-enrichment libraries that were ultra-deep sequenced in regions that previously have been associated with AD. The selected regions correspond to 0.1% of the genome. Using the raw calls from four different somatic mutation callers and strict downstream filtering, we were able to validate one rare somatic mutation in the brain of a LOAD patient at an alternative allele frequency (AAF) of 0.4%. This specific variant was found to affect transcription factor binding sites upstream of the gene *CD55*, a gene that is a regulator for the complement system.

## Results

### Deep sequencing and somatic mutation calling

To assess the presence of tissue-specific mutations in the brain, we obtained blood and frozen temporal cortex samples from EOAD (*n* = 4) and LOAD (*n* = 4) patients, as well as aged- and gender-matched non-AD individuals (*n* = 8) (Supplementary Material, Table S1).

We created targeted capture libraries containing 11 genomic regions covering 2.86 Mb, which harbor 28 genes (Supplementary Material, Table S2). Five of the regions contained genes associated with AD in a large meta-analysis (1). If the genes were located in a gene cluster, neighboring genes were included. In addition, previously known AD genes (*APP*, *PSEN1*, *PSEN2* and *APOE*) were also included. All selected regions contained large upstream and downstream area to include regulatory regions.

Sequencing libraries were created for each sample (*n* = 32; Fig. 1A) using 4 μg of genomic DNA to create the sample libraries (1.5–6.5 μg) that were hybridized to the targeted array (Supplementary Material, Table S3). Two libraries (blood sample 7 and brain sample 16) were excluded from further analysis along with their respective sample pair due to an insufficient number of reads (<8 million reads) resulting in low coverage and complexity (Supplementary Material, Figs S1 and S2). The remaining 14 sample pairs had an average sequencing depth of 698× ± 23× (mean ± standard error of the mean (SEM)) across the captured targets, where 85.5 ± 0.6% of the sequence from each individual had a minimum of 50× coverage (Supplementary Material, Fig. S1A and B and Table S3). This experimental workflow resulted in ultra-deep coverage, low frequency of duplicate reads and good complexity, enabling us to detect tissue-specific mutations in the temporal cortex (Supplementary Material, Fig. S1C and D).

**Figure 2:**
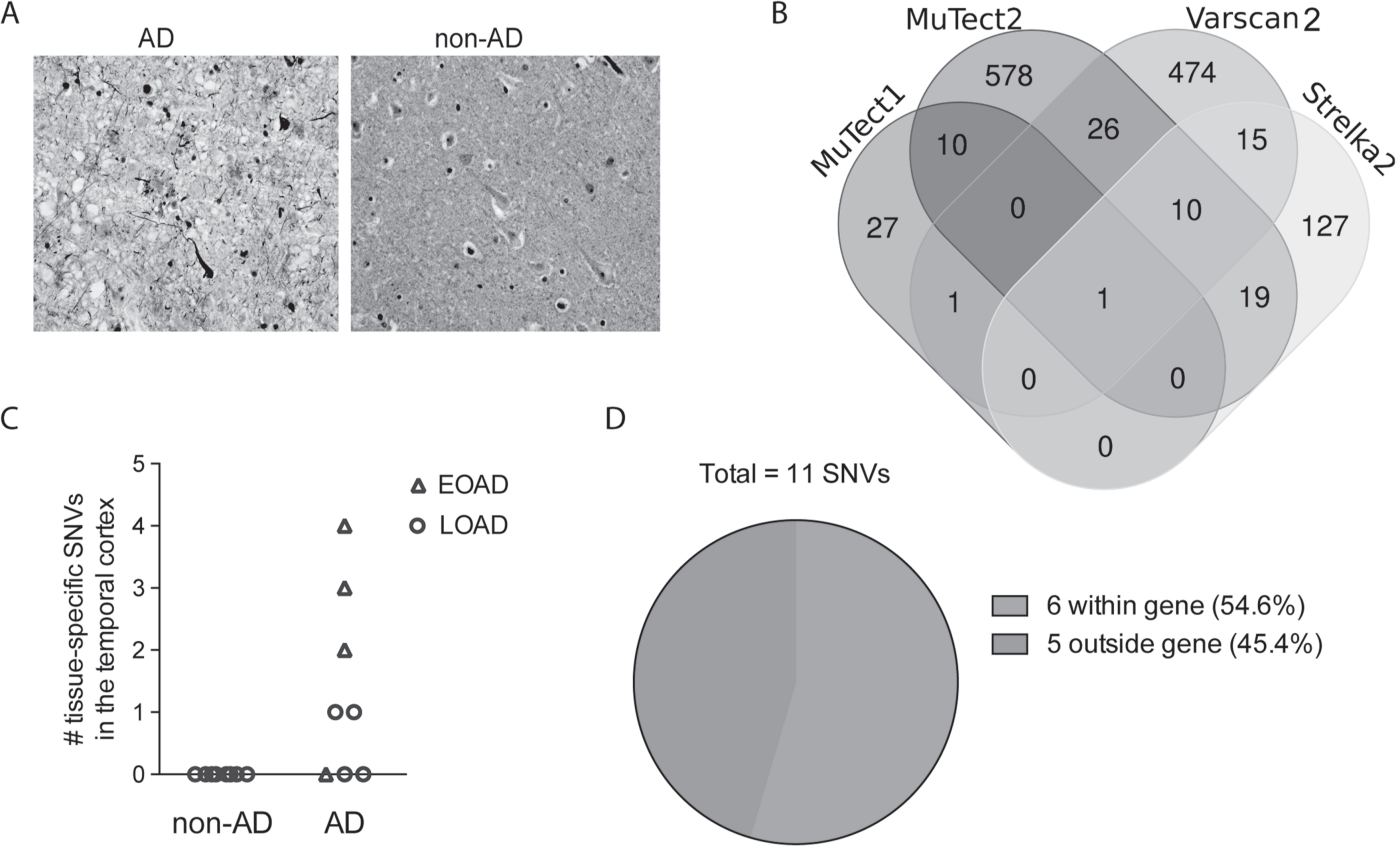
Tissue-specific SNVs in the brain detected in AD patients. (**A**) Bielschowsky-stained frontal cortex from AD and non-AD individuals. AD patients show signs of plaques and tangles. (**B**) Overview of SNVs called by different somatic callers. SNVs identified by minimum three somatic callers were considered possible brain-specific SNVs. (**C**) In total, 11 tissue-specific SNVs were detected in the brain of the AD patients while no SNVs were detected in non-AD, and more SNVs were detected in EOAD than in LOAD. (**D**) Although majority of the regions targeted were intergenic, more SNVs were detected within genes.

The libraries with the lowest input (1–2 μg) had higher duplication rate [26 ± 3.7% (mean ± SEM)], lower complexity (0.75 ± 0.4, ratio expected and observed molecules) and less coverage (422× ± 108×), compared with the higher input (2.5–6.5 μg) libraries (7 ± 0.6%, 0.93 ± 0.01, 682× ± 31×, for duplication, complexity and coverage, respectively) (Supplementary Material, Fig. S2). Based on our experimental setup, we can conclude that 2.5 μg of the sample library is sufficient to achieve the complexity and sequencing depth required to identify tissue-specific mutations in a bulk tissue preparations (Supplementary Material, Fig. S2 and Table S3).

**Table 1:** Tissue-specific SNVs identified in the brain DNA

				MuTect1				MuTect2				Strelka2				Varscan2			
				Blood		Brain		Blood		Brain		Blood		Brain		Blood		Brain	
Group sample	SNV	Locus	Location	Ref/Alt	AAF (%)	Ref/Alt	AAF (%)	Ref/Alt	AAF (%)	Ref/Alt	AAF (%)	Ref/Alt	AAF (%)	Ref/Alt	AAF (%)	Ref/Alt	AAF (%)	Ref/Alt	AAF (%)
LOAD 1/2	chr1:207351003C>A	CR1	Intergenic	na	na	na	na	423/0	0	546/8	1.2	425/0	0	586/8	1.3	424/0	0	584/7	1.2
LOAD 19/20	chr1:207461994C>T	CR1	Upstream *CD55*	794/0	0	582/6	1.0	1472/0	0	997/10	1.0	1476/0	0	1002/10	1.0	1477/1	0	1001/10	1.0
EOAD 27/28	chr1:207550332T>C	CR1	Intergenic	na	na	na	na	697/0	0	398/6	1.6	701/0	0	400/6	1.5	700/0	0	400/5	1.2
EOAD 27/28	chr1:227069718G>T	PSEN2	Missense *PSEN2*	na	na	na	na	491/0	0	290/5	1.3	490/0	0	289/5	1.7	490/0	0	287/5	1.7
EOAD 25/26	chr2:128054946G>T	BIN1	Upstream *ERCC3*	na	na	na	na	403/0	0	391/5	1.0	409/0	0	397/5	1.2	409/0	0	396/5	1.2
EOAD 11/12	chr8:27316070C>A	CLU/PTK2B	3′UTR *PTK2B*	na	na	na	na	332/0	0	414/6	1.0	334/0	0	417/6	1.4	330/0	0	413/6	1.4
EOAD 25/26	chr11:121250381G>T	SORL1	Intergenic	na	na	na	na	394/0	0	467/7	1.0	394/0	0	473/7	1.5	393/0	0	472/7	1.5
EOAD 11/12	chr11:121363100C>A	SORL1	Intron *SORL1*	na	na	na	na	609/1	0	209/6	2.6	614/1	0	222/6	2.6	612/0	0	221/6	2.6
EOAD 27/28	chr11:121401561A>G	SORL1	Intron *SORL1*	na	na	na	na	880/0	0	391/6	1.1	882/0	0	392/6	1.5	876/0	0	389/6	1.5
EOAD 11/12	chr21:27421506G>T	APP	Intron *APP*	na	na	na	na	696/0	0	371/5	1.0	704/0	0	428/5	1.1	697/0	0	427/5	1.2
EOAD 11/12	chr21:27489758G>A	APP	Intron *APP*	na	na	na	na	987/0	0	579/6	0.7	987/0	0	594/6	1.0	979/0	0	585/6	1.0

Sample ID is according to Supplementary Material, Table S1; chromosomal position is according to hg19. For the sequencing results, the number of reads for reference (Ref) and alternative (Alt) allele are reported, as well as the AAF for the brain and the blood. na means not available.

**Table 2:** Comments from the somatic variant callers on the tissue-specific SNVs in brain

Group sample	SNV	MuTect1	MuTect2	Strelka	Varscan2
LOAD 1/2	chr1:207351003C>A	fstar_tumor_lod, possible_contamination	t_lod_fstar	LowEVS	PASS
LOAD 19/20	chr1:207461994C>T	possible_contamination	PASS	LowEVS	PASS
EOAD 27/28	chr1:207550332T>C	possible_contamination	PASS	LowEVS	PASS
EOAD 27/28	chr1:227069718G>T	fstar_tumor_lod, possible_contamination	t_lod_fstar	LowEVS	PASS
EOAD 25/26	chr2:128054946G>T	fstar_tumor_lod, possible_contamination	t_lod_fstar	LowEVS	PASS
EOAD 11/12	chr8:27316070C>A	fstar_tumor_lod, possible_contamination	t_lod_fstar	LowEVS	PASS
EOAD 25/26	chr11:121250381G>T	fstar_tumor_lod, possible_contamination	t_lod_fstar	LowEVS	PASS
EOAD 11/12	chr11:121363100C>A	fstar_tumor_lod, possible_contamination	t_lod_fstar	PASS	PASS
EOAD 27/28	chr11:121401561A>G	fstar_tumor_lod, possible_contamination	t_lod_fstar	LowEVS	PASS
EOAD 11/12	chr21:27421506G>T	na	t_lod_fstar	LowEVS	PASS
EOAD 11/12	chr21:27489758G>A	fstar_tumor_lod, possible_contamination	t_lod_fstar	LowEVS	PASS

Sample ID is according to Supplementary Material, Table S1; chromosomal position is according to hg19. For the SNVs, the comment from default setting of every caller is showed.

To identify rare tissue-specific mutations in bulk DNA, robust downstream filtering was needed (Fig. 1B). Four different somatic mutation callers, MuTect v1, MuTect v2, Strelka2 and Varscan2, were used to identify mutations in every sample pair. To be able to identify tissue-specific mutations in the brain, the blood sample was used as a reference to eliminate germline mutations and artifacts. Instead of using the callers’ default somatic filtering, all calls were kept, and instead a downstream somatic SNV filtering was applied (see Materials and Methods). Variants that were identified in the brain sample but not in the blood by all the four callers were considered as tissue-specific mutations in the temporal cortex, and variants identified by at least three of four callers were considered as possible temporal cortex tissue-specific mutations.

### Allelic imbalance was observed during library preparation and/or sequencing

Due to the deep mean coverage, we expected true heterozygote SNVs to show an AAF between 40 and 60%. However, discrepancy in the AAF between the brain and blood DNA of the same individual was noted for several SNVs. Since the DNA input used for the hybridization to the library was higher than recommended, the observed allelic imbalance could be related to that. However, the allelic imbalance was more frequent for the brain samples, where mutations carrying 30–40% AAF in blood, showed around 20% AAF in the brain (Supplementary Material, Fig. S3), although most often the blood libraries had higher input than the brain libraries (Supplementary Material, Table S3).

To further analyze this, one variant, rs2298813, was selected to be tested using droplet digital PCR (ddPCR) assay (Supplementary Material, Table S4). Deep sequencing had revealed that the variant was heterozygous with 45% AAF in the brain tissue sample and 27% in the blood. However, the results from the ddPCR assay showed the presence of the mutant allele to be ~ 50% in both tissues (Supplementary Material, Table S4). In addition, another variant, rs73082760 (chr1:207911130G>A), showed allele frequencies of 16.7% in brain and 4.5% in blood in the sequencing data. However, when validating the variant with ddPCR the allele frequencies were similar in brain and blood (AAF of 25.9 and 29.1%, respectively) (Supplementary Material, Table S4). Further analysis of samples collected during the different steps in the library preparation showed that the allelic imbalances appear during the capturing or amplification steps of the captured library since the imbalance was absent in previous steps of the library preparation (Supplementary Material, Table S4).

### Tissue-specific SNVs in the temporal cortex of AD patients but not in non-AD individuals

The EOAD patients were diagnosed around the age of 50 years and died before the age of 70, while LOAD patients were diagnosed after the age of 65 and died around the age of 80–90 years. All the AD patients were neuropathologically confirmed with AD (Fig. 2A; Supplementary Material, Table S1).

After applying the somatic filter (Fig. 1B and Materials andMethods), we had in total 39, 644, 172 and 527 potential brain-specific SNV calls from MuTect1, MuTect2, Strelka2 and Varscan2, respectively. In total, 1288 potential brain-specific SNVs were identified in the sequencing data (Fig. 2B). One SNV was detected by all four callers and was considered to be a true tissue-specific somatic mutation. Ten SNVs were identified by three callers, and these were considered to be possible tissue-specific mutations (Table 1). The SNVs detected by 2 callers (71 SNVs, see Supplementary Material, Table S5) or by 1 caller (1206 SNVs) were not considered to be brain-specific mutations.

The 11 SNVs that were identified by at least 3 of the callers were rare in DNA from temporal cortex with an average 1% AAF (0.7–2.6%), but absent in blood DNA. The default settings of the somatic callers failed to identify most of them (Table 2). This emphasizes the importance of using different settings than the default filtering when identifying rare somatic mutations in bulk DNA.

The SNVs were only detected in brain tissue from patients with AD: two were identified in LOAD patients and nine in EOAD patients (Table 1 and Fig. 2C). Although the majority of the genomic regions included in the study were outside genes, more variants were detected within genes (6 out of 11 SNVs) (Table 1 and Fig. 2D).

### Validation of the somatic SNVs identified in DNA from AD brains using ddPCR

To confirm the tissue-specific mutations and to compare mutations called by different sets of somatic callers, we selected 14 SNVs and performed rare event detection using ddPCR. One SNV was detected by all four somatic callers, three by MuTect2, Strelka2 and Varscan2, one by MuTect1 and Varscan2 and nine SNVs by MuTect1 and MuTect2 (Table 3). Assay design or optimization failed for eight of the SNVs, either due to the complexity of the sequence region or technical limitations with the ddPCR system (Table 3).

**Table 3:** Validation of tissue-specific SNVs in brain using ddPCR

				Brain Validation				Blood Validation			
Group sample	SNV	Locus	Somatic caller	Fractional abundance (95% CI) [a/(a + b)%]	Total DNA	Total haploid copies	Proportion mutant allele, 1 in X	Fractional abundance (95% CI) [a/(a + b)%]	Total DNA	Total haploid copies	Proportion mutant allele, 1 in X
LOAD 19/20	chr1:207461994C>T	CR1	a	0.39 (0.31;0.47)	151	45 740	256	0	167	50 725	0
LOAD 1/2	chr8:26930598T>C	CLU/PTK2B	b	0.106 (0.061;0.145)	215	65 268	943	0.097 (0.05;0.15)	96	28 988	1031
NL_Old 21/22	chr2:127905153G>T	BIN1	b	0	88	20 287	na	na			
NL_Old 21/22	chr11:121034807G>T	SORL1	c	0	84	19 288	na	na			
EOAD 25/26	chr11:121332780G>T	SORL1	b	0	12	3681.8	na	na			
NL_Old 23/24	chr11:121415560G>T	SORL1	b	0	34	10 060	na	na			
LOAD 19/20	chr11:121382085G>T	SORL1	b	Assay failure				Assay failure			
NL_Old 21/22	chr1:207586559G>T	CR1	b	Design fail				Design fail			
EOAD 25/26	chr2:128054946G>T	BIN1	d	Design fail				Design fail			
EOAD 25/26	chr11:120914367G>T	SORL1	b	Design fail				Design fail			
NL_Old 21/22	chr11:121256750G>T	SORL1	b	Design fail				Design fail			
EOAD 11/12	chr11:121436267G>T	SORL1	b	Design fail				Design fail			
EOAD 25/26	chr11:121250381G>T	SORL1	d	Design fail				Design fail			
EOAD 11/12	chr11:121363100C>A	SORL1	d	Design fail				Design fail			

Values are given as fractional abundance with 95% CIs. Total DNA and haploid copies are given, as well as the calculated proportion of the mutant allele in the tissue.

Old non-AD individual (NL_Old). Sample ID is according to Supplementary Material, Table S1, chromosomal position is according to hg19 and locus is according to Supplementary Material, Table S2. Somatic sequencing callers: a, MuTect1, MuTect2, Strelka2, Varscan2; b, MuTect1, MuTect2; c, MuTect1, Varscan2; d, MuTect2, Strelka2, Varscan2.

For the remaining six variants, four variants were not detected in the temporal cortex. Those variants were very rare; the AAF was between 0.4 and 0.8% in the sequencing depending on the somatic caller (Supplementary Material, Table S5). The fifth variant, chr8:26930598 (AAF of 0.6–0.8% in the sequencing by MuTect2 and MuTect1), was detected with ddPCR in the temporal cortex at 0.1%; however, it was detected at the same allele frequency in blood (Table 3).

**Figure 3:**
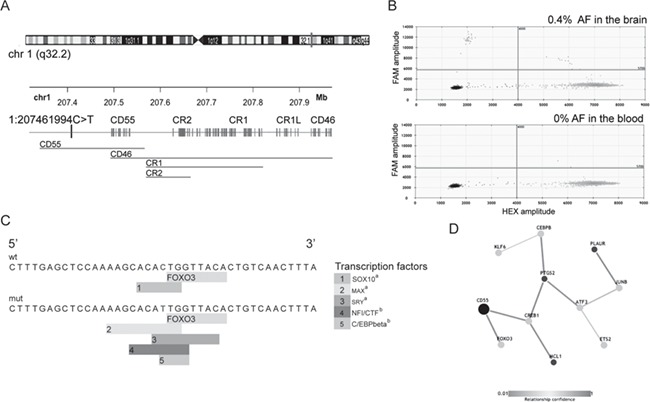
SNV detected by all somatic callers and validated using ddPCR. (**A**) Overview of the CR1-locus selected on the library, covering five genes. A brain-specific SNV was identified in this region where chr1:207461994C>T was detected in the brain by ultra-deep sequencing. GeneHancer Regulatory Elements and Gene Interactions showed that the SNV is located in a regulatory region of the gene CD55. (**B**) The identification of chr1:207461994C>T by ultra-deep sequencing was validated with ddPCR to be present in DNA from temporal cortex and was absent in blood. (**C**) Predicted transcription factor binding sites in the DNA sequence surrounding chr1:207461994C>T for the wild-type allele (C allele) and the mutant allele (T allele). a, JASPAR; b, PROMO. (**D**) Transcriptional regulation of the *CD55* gene is regulated by the FOXO3 transcription factor (data from PathwayNet).

The SNV, chr1:207461994C>T (AAF of 1% in the sequencing), is located in the CR1-region (Fig. 3A). It was detected by all four somatic callers and validated using ddPCR with a fractional abundance of 0.4% in DNA from the temporal cortex but not present in blood DNA (Table 3 and Fig. 3B).

### The somatic mutation impairs transcription factor binding sites upstream of the CD55 gene

The validated SNV, chr1:207461994C>T, was detected in a LOAD patient, a man that was diagnosed with AD at the age of 70 and died at the age of 89 years (Supplementary Material, Table S1), but not detected in other samples (Supplementary Material, Table S6). The variant was located 32 kb upstream of the gene coding for CD55 and was situated within a candidate regulatory region of the gene (Fig. 3A). *In silico* analysis of the genome sequence around the SNV for each allele was carried out to search for effects on transcription factor binding sites. The results revealed that in the sequence containing the mutant allele (T-allele), the SOX10 transcription factor binding site is lost, while four new binding sites for the MAX, SRY, NFIC and CEBPFβ transcription factors were introduced (Fig. 3C; Supplementary Material, Table S7). In addition, the SNV is located immediately upstream of the FOXO3 transcription factor binding site and could possibly impair its binding, with direct impact on the *CD55* transcriptional activity (Fig. 3D).

## Discussion

In this study, we used ultra-deep sequencing of 2.86 Mb of genomic regions to identify somatic mutations in human temporal cortex from both 4 early and 4 LOAD patients as well as 6 non-AD individuals. We analyzed 0.1% of the human genome that cover regions that previously have been associated with AD and genes that have shown to be mutated in AD. In addition to coding regions, we also covered non-coding genomic regions that potentially could harbor regulatory elements. A recent study using single-cell DNA sequencing has shown that tissue-specific mutations exist in the brain where every neuron has up to 1500 somatic mutations (5). Identifying somatic mutations in bulk DNA is still quite challenging and it is technically limited to studies of only parts of the genome (3,6–9). Recent studies on AD brain samples have determined few brain-specific mutations, where two brain-specific SNVs were identified by deep sequencing of the coding region in 11 genes using 100 AD brain samples (6), and 5 brain-specific SNVs were identified by deep sequencing of the exons of 56 genes in 20 AD brain samples (3). Another study analyzed the genomic regions of 4 genes in 72 AD brain samples, including 10 kb upstream and downstream regulatory regions, and identified 2 somatic mutations in the coding region of *MAPT* (7).

Here we used high DNA input and tailored downstream bioinformatics analysis and achieved the required complexity and sensitivity to identify 11 potential brain-specific variants. All 11 variants were detected in DNA from AD brains, where 9 variants were observed in EOAD patients. The lack of brain-specific variants in non-AD individuals could indicate that our filtering strategy was too strict, or that brain-specific variants in non-AD are below the level of detection using the method in this study. Ten of the variants were detected by three of the four somatic callers, but one variant 32 kb upstream of the *CD55* gene was identified by all four callers. The variant, chr1:207461994C>T, was further validated with ddPCR at an allele frequency of 0.4% in the temporal cortex of the LOAD patient. Even though the SNV is not located within a coding region, it is positioned within a candidate regulatory region of the gene *CD55*. Considering that the genetic causes of AD are largely unknown, variants in regulatory regions could play a role in the disease etiology (13). CD55 is involved in the regulation of the complement system where it binds to C3b and C4b, thereby affecting the formation of the C3 convertase. Increased expression of CD55 and inhibition of complement activation leads to reduced tissue damage (14). CD55 is expressed in neurons during chronic inflammation to protect the neurons against the complement system (14,15), but it is also expressed in other brain cell types such as the glial cells (16). The complement system has been linked to AD, where the expression of the complement components is increased in the brains of AD patients, specifically in the affected sites (17,18), whereas the expression of the complement system regulators remains the same or is only slightly increased (19). In addition, CD55 has another role in the inflammatory system where it inhibits natural killer cells and together with CD97 promotes B and T cell proliferation (20). B cells are known to secrete antibodies that detect the Aβ peptide, and T cells have been detected near plaques in human AD brains (21,22). Studies on animal models have shown that B and T cells have important roles in the pathogenesis of AD (21,22). Therefore, a variant located within the regulatory region of the *CD55* gene could contribute to misregulation of the protein, leading to increased activity of the complement system and increased tissue damage.

Analysis of the mutant allele of chr1:207461994 showed that this specific SNV affects the binding sites for several transcription factors. The binding site for the SOX10 transcription factor is lost, whereas other binding sites were introduced by the mutation. The SOX10 transcription factor is expressed in several tissues, including in the brain, where it is predominantly expressed in glial cells contributing to their development and maturation (23). The absence of the SOX10 transcription factor could lead to a reduced expression of CD55 followed by an increased activation of the complement system and an increased cell death. In addition, the SNV is located right next to the FOXO3 binding site that is known to interact with the *CD55* gene. This variant could affect the binding site of FOXO3 and consequently affect the expression of CD55, therefore contributing to the progression of AD.

It should be noted that the methods used in this study have several limitations. The sample set used in this study was small, and only part of the genome was analyzed. In addition, we analyzed bulk tissue from the temporal cortex, and therefore we cannot say what cell types are affected or if our finding is representative for other parts of the brain.

One of the characteristics of AD is the loss of neurons. Subsequently the DNA is lost along with the somatic mutations that could be the underlying reason for the neuron loss. This could affect the possibility of detecting causal variants in the bulk DNA. Furthermore, the brain samples are obtained post-mortem, and the time from death to biopsy may result in DNA fragmentation, which can affect the variant allele frequency making somatic variant detection difficult. In addition, somatic variant callers have different sensitivity and specificity and are designed to call variants in cancer tissues at certain depth (~100× coverage). In the cases of higher coverage, the default somatic filters applied by the callers become too strict. The variants are often very rare, and in cases of >1000× coverage, the callers lack sensitivity and specificity. Despite deep coverage, we detected many false-positive calls. We could exclude many of them using strict downstream filtering and four different somatic callers; nevertheless, it is possible that during the filtering we have excluded true positive variants.

We noticed that the sequencing libraries were biased toward the reference nucleotide, where heterozygous variants showed lower AAF in brain tissue compared to blood. Further analysis on variants in the different steps of the library preparation indicated that the allelic imbalance occurred during the capturing and/or post-capturing amplification step. Although we did not detect fragmentation of the DNA from the brain samples, we cannot rule out the possibility that smaller fragments were present in the bulk DNA but were cleaned out during the library preparation. The quality of the sample is important for the outcome of the library preparation, as it might affect downstream applications. The ddPCR assays cover smaller genomic regions (60–70 bp) and are possibly better to use to assess the mutation frequency in degraded DNA, compared to sequencing, which needs longer insert sizes (125 bp).

In conclusion, we show that somatic mutations occur in the brain and can be detected at low frequency, at 0.4%, in bulk DNA using ultra-deep sequencing. However, in order to do so one would need high DNA input and comprehensive bioinformatic downstream analysis. The variant we validated may contribute to AD by interfering with the regulatory component of the complement system. However, further studies are needed to fully understand the impact of this mutation and others on the neuronal loss and the disease process. Our study emphasizes the need for additional studies of somatic mutations in aging and age-associated disease, including AD, to gain further knowledge on their molecular genetic mechanisms.

## Materials and Methods

### Samples

EOAD patients (*n* = 4), LOAD patients (*n* = 4) from the Brain Bank at Karolinska Institutet, Sweden, and aged- and gender-matched non-AD (*n* = 8) individuals from the Netherlands Brain Bank (NBB) were included in the study (Supplementary Material, Table S1). For every individual, we obtained both frozen brain (temporal cortex) and blood tissue samples. All AD patients were clinically and neuropathologically confirmed as definitive AD. Non-dementia individuals were clinically and neuropathologically confirmed to not have AD.

All participants (or a next of kin acting as proxy) gave informed consent to participation in genetic studies and to brain donation. The informed consent forms and study protocols were approved by the local ethics committees and conform to the Helsinki Declaration.

### DNA extraction

The DNA from whole blood (EDTA) and frozen brain was extracted using Gentra Puregene tissue and blood kits (Qiagen, Hilden, Germany) as recommended by the supplier. The frozen brain tissue was grinded to powder and added to a cell lysis solution and with Proteinase K as recommended by the supplier. The solution was incubated overnight at 55°C. The concentration of the DNA was measured using Qubit dsDNA assay kit.

### Regions selected on the DNA targeting array

Eleven regions associated to AD, through candidate studies or meta-analysis (1), were selected for target enrichment (Supplementary Material, Table S2). In total, the regions included exons and introns of 28 genes, and intragenic regions covering 2.86 Mb (Supplementary Material, Table S2). Probes for the targeted regions on the SeqCap EZ Choice library (Roche, Basel, Switzerland) were designed using the software NimbleDesign from Roche.

### Sample library preparation

The DNA was fragmented to an average fragment size of 250 bp using Covaris S220 (180 s, 10% duty factor, peak power = 175 W, 200 cycle/burst). In total, 6 μg of DNA was sonicated in 130 μl of distilled water, and then each sample library was made using 4 μg of DNA (that represent 606 060 cells and 6.6 pg DNA/cell) as starting material. End repair was performed using 10× T4 DNA ligase buffer with 10 mm ATP, 10 mm dNTP mix, T4 and Klenow DNA polymerases and T4 polynucleotide kinase. To add A-bases to the 3′ end of the DNA fragments 10× Klenow buffer, dATP and Klenow exo− were used (all reagents from New England Biolabs, Ipswich, Massachusetts, USA). Adapters from Roche were ligated in next step with 2× Quick ligase buffer and quick T4 DNA ligase. The size selection of the products was done using agarose gels and followed by enrichment of the sample library by PCR, using primers (5′-AATGATACGGCGACCACCGAGA-3′ and 5′-CAAGCAGAAGACGGCATACGAG-3′), 5× Phusion HF buffer, dNTP mix and Phusion DNA polymerase (New England Biolabs, Ipswich, Massachusetts, USA). The PCR protocol was as follows: 2 min 98°C, 8 cycles of 30 s at 98°C, 45 s at 65°C and 30 s at 72°C, then 5 min at 72°C and finally hold at 4°C. In every step, the purification was performed using Agencourt Ampure XP beads. The amount of each sample library was quantified and average base pair size estimated using the Bioanalyzer 2100.

### Capturing on DNA targeting array

For each sample library, we tried to maximize the DNA amount to increase the depth. The amount of sample library used ranged between 1.5 and 6.5 μg and was individually captured on the library according to provided protocol from manufacturer. Specificity of the capturing was confirmed with qPCR for three genes included on the array and two genes that were not included. Each captured library was measured using the Bioanalyzer 2100 and pooled equally in two pools to be sequenced on two lanes at NGI Sweden, Science for Life Laboratories, Stockholm, on Illumina HiSeq-2500, 2 × 125 bp.

### Analysis

#### Quality control

Using FastQC, we could determine that all reads passed quality filters after sequencing. MultiQC (24) was used to aggregate the quality of the raw fastq files, and complexity analyzed using Preseq (25). The complexity ratio reported is the ratio of expected and observed molecules.

#### Analysis workflow

To remove possible adapter content, fastq files were trimmed using TrimGalore (https://www.bioinformatics.babraham.ac.uk/projects/trim_galore/) with default parameters for paired end sequence. Trimmed fastq files were then aligned to the human reference genome hg19 using BWA (26) with default parameters. Alignments were sorted and indexed using SAMtools 0.1.19 (27). For every sample, duplicated reads were marked using Picard (https://broadinstitute.github.io/picard/). Local realignment around indels was performed, and the two samples from the same individual were aligned together to improve variant calling using the Genome Analyzer Toolkit v3.4.0 (GATK) (28). Variants were called using the GATK-HaplotypeCaller and the somatic mode of four somatic callers: MuTect (v. 1.1.5) (29), GATK-MuTect2, Strelka2 (v. 2.9.3) (30) and Varscan2 (v. 2.3.7) (31) with brain as tumor and blood as normal. Variants with the comments `clustered events’, `poor mapping region’, `nearby gap’, `triallelic sites’ and `strand artifact’ from the MuTect callers were excluded. All SNVs in segmental duplicated regions (UCSC genome regions) (32,33) were excluded. Annotations were done using snpEff_4.2 (34).

#### Somatic filtering

SNVs detected by each somatic variant caller were analyzed and filtered to identify somatic SNVs (Fig. 1B).

The criteria used to identify somatic mutations were the following:

i) SNVs with 50× minimum read depth in all samples were included.
ii) SNVs with minimum 5 alternative allele depth in the brain were included.
iii) SNVs with AAF < 20% in both tissues were included.
iv) SNVs with brainAAF/bloodAAF ratios between 0.8 and 1.2 were excluded.
v) SNVs with significant difference between the brain read count and the blood read count (*P* < 0.05, Fisher’s exact test) were included.
vi) Recurrent SNVs were excluded from the analysis.
vii) SNVs identified by at least three of the four callers were considered possible somatic variants.

#### Analysis of regulatory regions and transcription factor binding sites

Candidate enhancers in the sequence were retrieved from GeneHancer (35) and visualized in the UCSC genome browser. The prediction of transcription factor binding sites was performed using the software PROMO v3.0.2 (36,37) and JASPAR v5.0_ALPHA (38). The interaction between CD55 and FOXO3 was analyzed using PathWayNet (39).

####  ddPCR validation

Primer-probe assays were designed and ordered from BioRad’s web interface for rare event detection assay design. Raw fluorescence data for each well were analyzed and exported from the manufacturer’s software (QuantaSoft version 1.6, Bio-Rad, Hercules, California, USA). Each assay measurement comprises data from 2 merged wells where a minimum of 10 ng DNA was analyzed. The data from replicate ddPCR wells were merged and the combined droplet counts used. Sample data were only accepted when falling within established detection parameters, which include a minimum of 3 positive droplets per sample and 10 000 accepted droplets per well. The fractional abundance and Poisson-based 95% confidence intervals (CIs) were obtained from the QuantaSoft software.

## Supplementary Material

HMG2019_Helgadottir_SI_20190319_ddz085
