# Supplementary material for: Somatic mutation that affects transcription factor binding upstream of CD55 in the temporal cortex of a late-onset Alzheimer disease patient
Source: Hum Mol Genet. 2019 Apr 24;28(16):2675–85. doi: 10.1093/hmg/ddz085 (PMC6688063; doi:10.1093/hmg/ddz085)
Supplement: HMG2019_Helgadottir_SI_20190319_ddz085 [file hmg2019_helgadottir_si_20190319_ddz085.pdf]

SUPPLEMENTARY MATERIAL

Somatic mutation that affects transcription factor binding upstream of *CD55* in the temporal cortex of a late-onset Alzheimer disease patient

Hafdis T. Helgadottir[1], Pär Lundin[2], Emelie Wallén Arzt[1], Anna-Karin Lindström[3,4], Caroline Graff [3,4] and Maria Eriksson[1*]

Affiliations:

[1]Department of Biosciences and Nutrition, Center for Innovative Medicine, Karolinska Institutet, Huddinge, Sweden. [2]Science for Life Laboratory (SciLifeLab), Stockholm University, Stockholm, Sweden. [3]Department of Neurobiology, Care Sciences and Society, Center for Alzheimer Research, Division for Neurogeriatrics, Karolinska Institutet, Solna, Sweden. [4]Unit for Hereditary dementias, Theme Aging, Karolinska University Hospital, Solna Sweden.

*Correspondence to:

Maria Eriksson, Department of Biosciences and Nutrition, Center for Innovative Medicine, Karolinska Institutet, Huddinge, Sweden; Tel: +46 852481066; Fax: +46 8311101 ; Email: Maria.Eriksson.2@ki.se

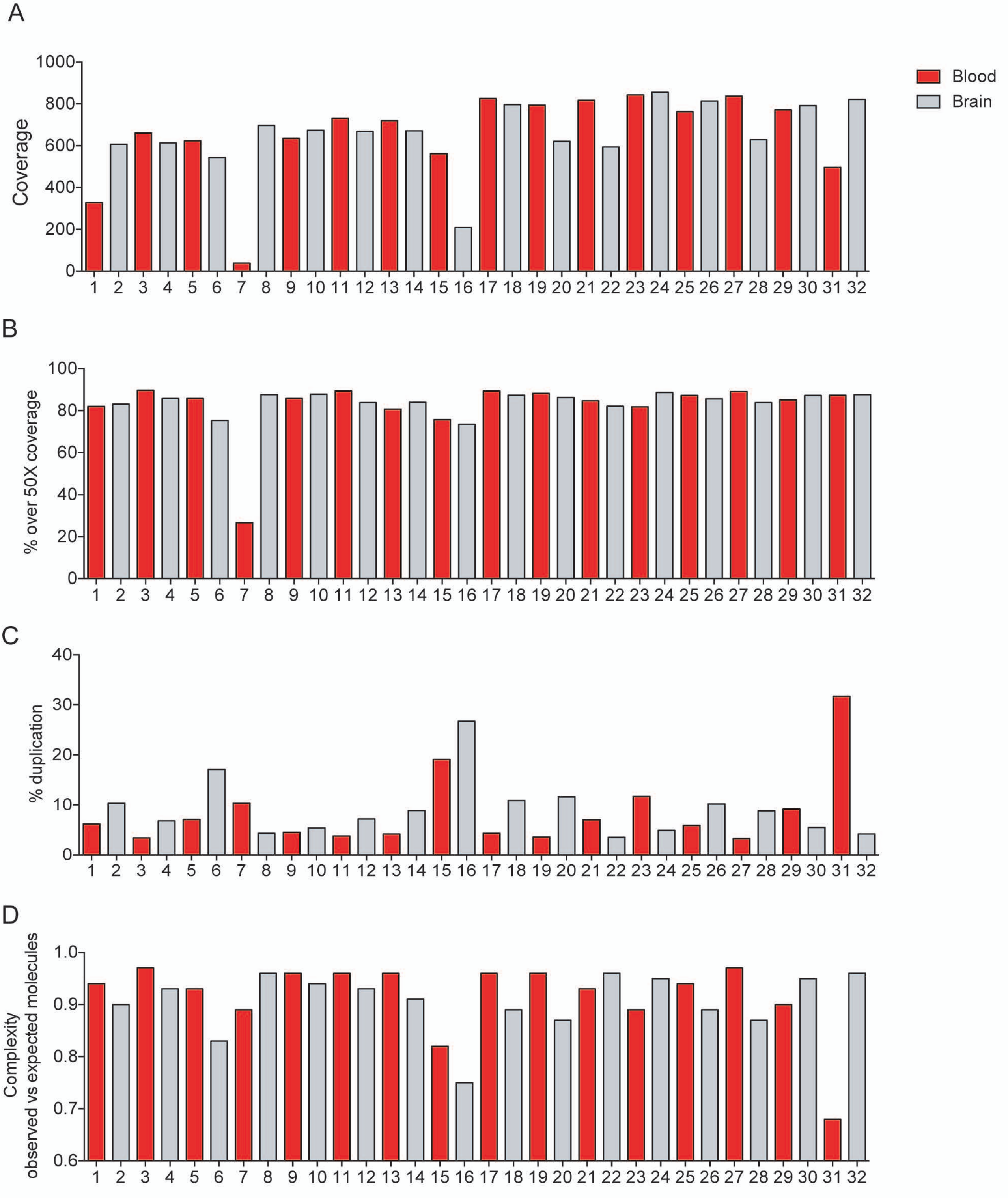

Supplementary Figure 1: Library quality. (A) Deep coverage was accomplished for all samples, apart from sample 7 and 16. This resulted in (B) minimum 50X coverage in 80% of the targeted regions, (C) low duplication reads and (D) good complexity (ratio of observed and expected molecules).

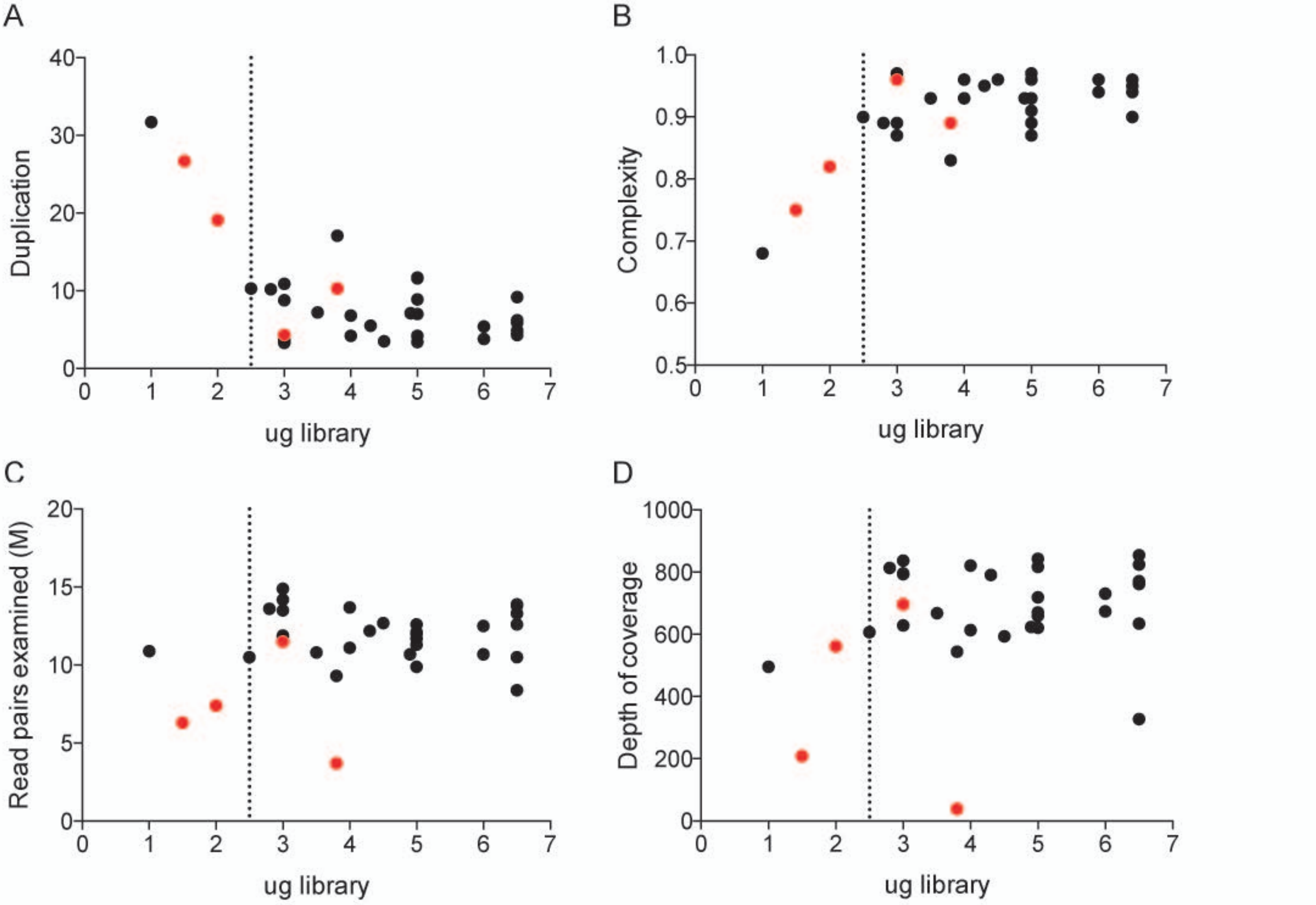

Supplementary Figure 2: High input resulted in good library quality. By using 2.5 ug of the sample library for the library preparation it was possible to get (A) low duplication rate, (B) high complexity, (C) around 10 M reads, that lead to (D) around 600x coverage of the 2.8 Mb targeted genome. Red dots indicate the two sample pairs that were excluded (sample 7-8 and 15-16).

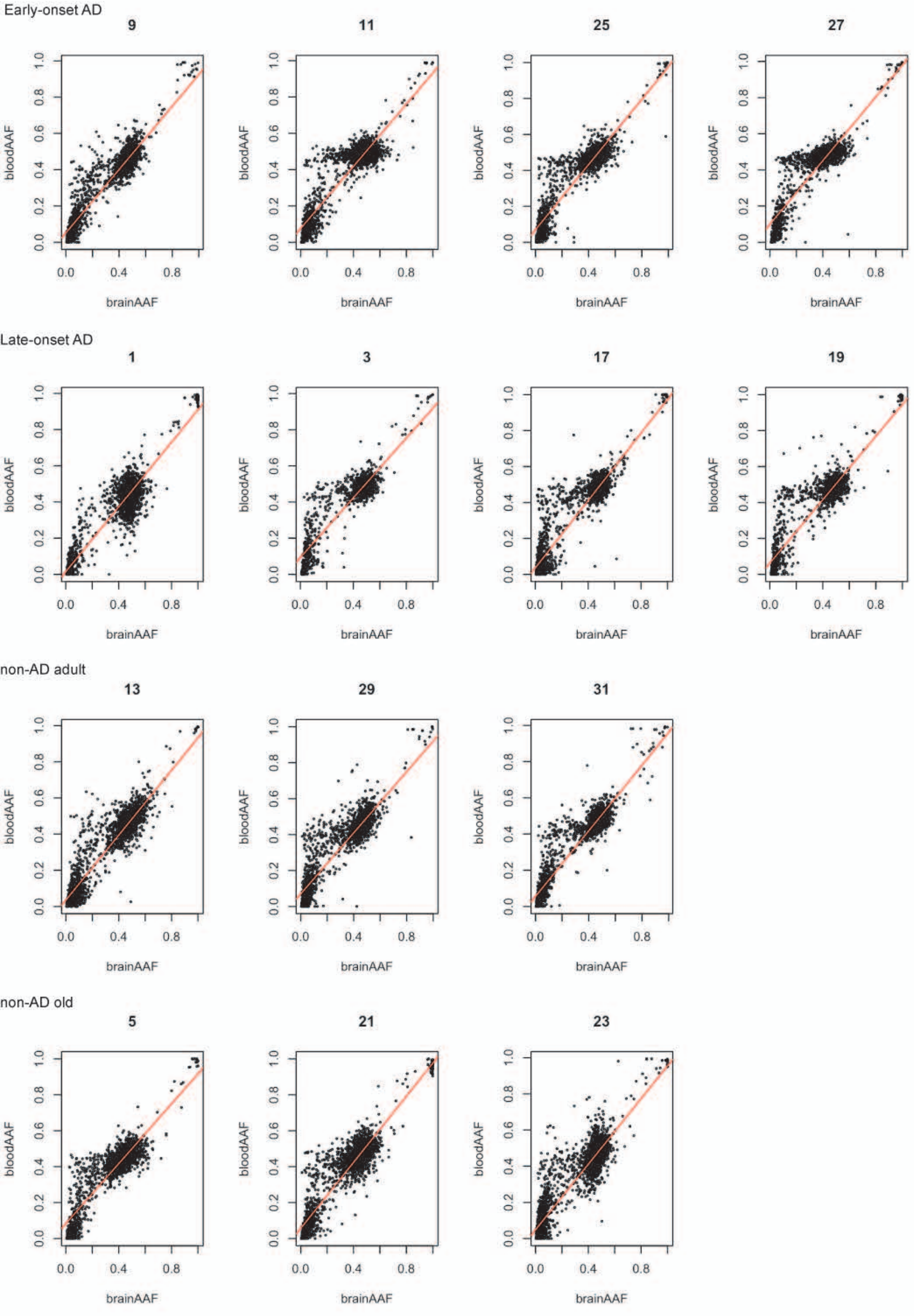
Early-onset AD
9
11
25
27
Late-onset AD
1
3
17
19
non-AD adult
13
29
31
non-AD old
5
21
23
bloodAAF
brainAAF
0.0
0.2
0.4
0.6
0.8
1.0

Supplementary Figure 3: Comparison on AAF in the blood (Y-axis) and brain (X-axis) showed allelic imbalance between the tissues. We detected in all individuals number of SNVs that showed ~30%-40% AAF in the blood but ~20%-30% AAF in the brain. Validation of one of the SNV that showed this pattern in the sequencing (AAF 45% and 27% in the blood and the brain respectively), was in fact heterozygote (~50%) both in blood and brain.

Table S1: Characteristic of the samples used in the study

		Sample ID	Gender	Age Onset	Age Death	APOE
Alzheimer´s disease	Early-onset	9/10	F	50	59	34
		11/12	F	49	65	33
		25/26	F	47	61	34
		27/28	M	52	68	34
	Late-onset	1/2	F	65	82	34
		3/4	F	65	79	34
		17/18	F	72	86	33
		19/20	M	70	89	34
Non-dementia	Adult	13/14	F	N/A	57	23
		15/16	F	N/A	53	33
		29/30	F	N/A	68	34
		31/32	M	N/A	70	34
	Old	5/6	F	N/A	84	34
		7/8	F	N/A	78	33
		21/22	F	N/A	85	33
		23/24	M	N/A	88	33

Four early-onset and four late-onset AD patients as well as 8 aged- and gender-matched non-dementia individuals were included in the study. For the sample ID the first number represent the blood sample and the second number represent the brain sample. Gender, age of onset and death, as well as the APOE alleles are showed in the table.

Table S2: Genomic regions included on SeqCap EZ Choice library

Locus	Covered	%of the library	% exons	Chr	Start position	Stop position	Gene	Source
**CR1**	**521 391**	**18.1**	**5.38**	**chr1**	**207 318 317**	**207 969 861**		**Meta-analysis**
					207 494 817	207 534 311	CD55	
					207 627 645	207 663 240	CR2	
					207 669 473	207 815 110	CR1	
					207 818 458	207 897 036	CR1L	
					207 925 383	207 968 861	CD46	
**BIN1**	**331 352**	**11.6**	**8.89**	**chr2**	**127 804 598**	**128 175 995**		**Meta-analysis**
					127 805 599	127 864 903	BIN1	
					127 941 412	127 963 343	CYP27C1	
					128 014 866	128 051 752	ERCC3	
					128 056 245	128 100 805	MAP3K2	
**CLU/PTK2B**	**744 501**	**26.1**	**4.49**	**chr8**	**26 722 922**	**27 534 286**		**Meta-analysis**
					27 092 840	27 115 956	STMN4	
					27 142 403	27 168 836	TRIM35	
					27 168 999	27 316 908	PTK2B	
					27 317 278	27 336 813	CHRNA2	
					27 348 519	27 402 439	EPHX2	
					27 454 434	27 472 328	CLU	
					27 491 577	27 530 537	SCARA3	
**PICALM**	**262 571**	**9.2**	**2.07**	**chr11**	**85 667 213**	**85 955 805**		**Meta-analysis**
					85 668 214	85 780 923	PICALM	
**SORL1**	**569 299**	**19.9**	**6.92**	**chr11**	**120 894 802**	**121 509 471**		**Meta-analysis**
					120 894 813	120 960 354	TBCEL	
					120 973 375	121 061 515	TECTA	
					121 163 577	121 184 119	SC5D	

					121 322 912	121 504 471	SORL1	
**PSEN2**	**30 221**	**1.1**	**11.86**	**chr1**	**227 053 272**	**227 084 804**		**Candidate gene**
					227 058 273	227 083 804	PSEN2	
**TREM2**	**10 436**	**0.4**	**9.82**	**chr6**	**41 125 243**	**41 135 924**		**Candidate gene**
					41 126 244	41 130 924	TREM2	
**PSEN1**	**75 624**	**2.6**	**10.64**	**chr14**	**73 598 142**	**73 691 399**		**Candidate gene**
					73 603 143	73 690 399	PSEN1	
**PLD3**	**28 817**	**1**	**13.82**	**chr19**	**40 849 331**	**40 885 397**		**Candidate gene**
					40 854 332	40 884 397	PLD3	
**APOE**	**8 850**	**0.3**	**29.67**	**chr19**	**45 404 005**	**45 413 652**		**Candidate gene**
					45 404 035	45 406 981	TOMM40	
					45 409 006	45 412 652	APOE	
**APP**	**274 621**	**9.6**	**2.37**	**chr21**	**27 251 860**	**27 548 446**		**Candidate gene**
					27 252 861	27 543 446	APP	

Eleven regions were selected for targeting. Of them were 5 regions, covering 21 genes that have been linked to AD in previously published meta-analysis. The remaining seven regions, covering 8 genes, were selected as a previously known AD-candidate genes. Chromosome, start and stop of every region, as well as start and stop of each gene as reported in hg19, is reported in the table.

Table S3: Characteristics of libraries generated

Source	Group	Sample	ug Sample library used	nM Finished Library	Read pairs examined (M)	Duplication (%)	Complexity	Average Coverage (X)	% bases >50X
Blood	LOAD	1	6.5	5.11	8.4	6.2	0.94	328	82
Brain	LOAD	2	2.5	59.44	10.5	10.3	0.9	607	83.1
Blood	LOAD	3	5	156.35	12	3.4	0.97	660	89.7
Brain	LOAD	4	4	110.06	11.1	6.8	0.93	614	85.8
Blood	non_AD old	5	4.9	44.65	10.7	7.1	0.93	624	85.8
Brain	non_AD old	6	3.8	10.67	9.3	17.1	0.83	544	75.4
Blood	non_AD old*	7	3.8	3.78	3.7	10.3	0.89	39	26.6
Brain	non_AD old*	8	3	129.82	11.5	4.3	0.96	697	87.7
Blood	EOAD	9	6.5	48.74	10.5	4.5	0.96	635	85.8
Brain	EOAD	10	6	40.17	10.7	5.4	0.94	674	87.9
Blood	EOAD	11	6	147.29	12.5	3.8	0.96	731	89.4
Brain	EOAD	12	3.5	81.48	10.8	7.2	0.93	668	83.9
Blood	non_AD adult	13	5	97	12.1	4.2	0.96	719	80.8
Brain	non_AD adult	14	5	78.25	9.9	8.9	0.91	671	84
Blood	non_AD adult*	15	2	22.63	7.4	19.1	0.82	562	75.7
Brain	non_AD adult*	16	1.5	6.3	6.3	26.7	0.75	209	73.5
Blood	LOAD	17	6.5	64.65	13.8	4.3	0.96	825	89.4
Brain	LOAD	18	3	96.95	14.2	10.9	0.89	796	87.4
Blood	LOAD	19	3	158.49	14.9	3.6	0.96	794	88.3
Brain	LOAD	20	5	83.26	11.7	11.6	0.87	621	86.3
Blood	non_AD old	21	5	63.92	12.6	7	0.93	817	84.7
Brain	non_AD old	22	4.5	34.24	12.7	3.5	0.96	594	82.1
Blood	non_AD old	23	5	30.28	11.3	11.7	0.89	843	81.9
Brain	non_AD old	24	6.5	51.44	13.9	4.9	0.95	855	88.7
Blood	EOAD	25	6.5	93.69	12.6	5.9	0.94	762	87.3

Brain	EOAD	26	2.8	69.13	13.6	10.2	0.89	814	85.6
Blood	EOAD	27	3	166.54	13.5	3.3	0.97	837	89.1
Brain	EOAD	28	3	15.01	11.9	8.8	0.87	629	83.9
Blood	non_AD adult	29	6.5	32.6	13.3	9.2	0.9	771	85.1
Brain	non_AD adult	30	4.3	116.14	12.2	5.5	0.95	791	87.3
Blood	non_AD adult	31	1	19.5	10.9	31.7	0.68	496	87.4
Brain	non_AD adult	32	4	111.17	13.7	4.2	0.96	821	87.7

Overview of the amount of DNA used to create each sample library and the amount of generated after capturing. This lead to an average 11 M reads, 8.8% duplication rate, and 657X coverage whereas 83% of the targeted genome were above 50X. The high DNA input generated high complexity libraries (ratio expected and observed molecules). *Libraries that failed quality control and were excluded from the study

Table S4: Allelic imbalance analysis in the library preparation

Group Sample	SNV	Method	Brain Sequencing Ref/Alt	Brain Sequencing AAF (%)	Blood Sequencing Ref/Alt	Blood Sequencing AAF (%)	Brain Validation Fractional Abundance [95%CI] (a/(a+b)%)	Brain Validation Total DNA	Brain Validation Total Haploid copies	Blood Validation Fractional Abundance [95%CI] (a/(a+b)%)	Blood Validation Total DNA	Blood Validation Total Haploid copies
LOAD 1/2	chr1:121393684 G>A	Sequencing	150/121	45	107/40	27						
		Stock					53.4 [52.9;53.9]	422	128000	50.4 [49.1;51.8]	28	8572
		Library - Pre-Capturing					51.7 [48.9;54.5]	9	2744	52.1 [50;54.1]	11	3472
		Library - Post-Capturing					51.3 [50.4;52.2]	493	149480	28.6 [27.8;29.4]	67	20336
LOAD 19/20	chr1:207911130 G>A	Sequencing	55/11	16.7	192/9	4.5						
		Stock					25.9 [25.2;26.7]	88	26520	29.1 [28.3;29.9]	74	22520
		Library - Pre-Capturing					26.1 [23.5;28.7]	23	6872	24.5 [22.7;26.1]	131	39720
		Library - Post-Capturing					22.27 [21.5;23]	686	207880	24.9 [24.5;25.4]	391	118560

Droplet digital PCR (ddPCR) assays were used to test different steps in the library preparation and showed that the allelic imbalance arise during the capturing or during the amplification of the captured library. LOAD (late-onset AD). Sample ID according to Table S1, chromosomal position is according to hg19. For the sequencing results the number of reads for reference (Ref) and alternative (Alt) allele is reported, as well as the alternative allele frequency (AAF) for the brain and the blood. Validation values are given as fractional abundance with 95% confidence intervals (CI). Total DNA and haploid copies are given.

Table S5: SNVs identified by two somatic mutation callers

			MuTect1				MuTect2				Strelka2				Varscan2			
			Blood		Brain		Blood		Brain		Blood		Brain		Blood		Brain	
Group Sample	SNV	locus	Ref/ Alt	AAF (%)	Ref/Alt	AAF (%)	Ref/ Alt	AAF (%)	Ref/ Alt	AAF (%)	Ref/ Alt	AAF (%)	Ref/ Alt	AAF (%)	Ref/ Alt	AAF (%)	Ref/ Alt	AAF (%)
EOAD 11/12	chr1:207425938 C>A	CR1	na	na	na	na	393/0	0	468/6	1.0	395/0	0	475/6	1.2	na	na	na	na
LOAD 17/18	chr1:207432220 C>G	CR1	na	na	na	na	1087/0	0	848/12	1.6	1089/0	0	851/11	1.3	na	na	na	na
LOAD 17/18	chr1:207457418 A>T	CR1	na	na	na	na	1433/2	0	1260/15	1.3	1434/3	0	1264/15	1.2	na	na	na	na
EOAD 27/28	chr1:207470623 G>T	CR1	na	na	na	na	1147/0	0	724/7	0.8	na	na	na	na	1143/0	0	724/7	1.0
LOAD 1/2	chr1:207572011 A>C	CR1	na	na	na	na	na	na	na	na	728/0	0	422/5	1.2	724/0	0	419/5	1.2
Non-AD_Old 21/22	chr1:207586559 G>T	CR1	808/0	0	767/5	0.60	976/0	0	979/7	0.6	na	na	na	na	na	na	na	na
EOAD 9/10	chr1:207624143 G>A	CR1	na	na	na	na	777/0	0	685/8	0.8	na	na	na	na	774/0	0	681/8	1.2
EOAD 11/12	chr1:207672332 T>A	CR1	na	na	na	na	na	na	na	na	549/2	0	278/5	1.8	546/1	0	276/6	2.1
Non-AD_Old 5/6	chr1:207676826 G>T	CR1	na	na	na	na	na	na	na	na	347/0	0	362/6	1.6	345/0	0	361/6	1.6
Non-AD_Old 5/6	chr1:207808385 C>G	CR1	na	na	na	na	334/0	0	132/5	2.8	342/0	0	135/5	3.6	na	na	na	na
LOAD 17/18	chr1:207966393 G>A	CR1	na	na	na	na	1355/0	0	1237/13	0.9	1357/0	0	1240/13	1.0	na	na	na	na
EOAD 11/12	chr1:207968866 G>T	CR1	na	na	na	na	1024/0	0	318/5	1.2	na	na	na	na	1028/0	0	371/5	1.3
LOAD 3/4	chr2:127834872 G>T	BIN1	na	na	na	na	na	na	na	na	379/0	0	359/5	1.4	374/0	0	354/5	1.4
Non-AD_Old 21/22	chr2:127867185 G>T	BIN1	na	na	na	na	654/0	0	681/6	0.7	na	na	na	na	680/0	0	687/7	1.0
Non-AD_Old 21/22	chr2:127878905 G>T	BIN1	961/1	0	570/5	0.90	1137/0	0	663/5	0.7	na	na	na	na	na	na	na	na
Non-AD_Old 21/22	chr2:127905153 G>T	BIN1	884/0	0	763/5	0.70	1390/0	0	1028/8	0.6	na	na	na	na	na	na	na	na
EOAD 27/28	chr2:127914159 G>T	BIN1	na	na	na	na	1024/0	0	629/6	0.8	na	na	na	na	1030/1	0	631/6	0.9
LOAD 3/4	chr2:127922998 G>T	BIN1	na	na	na	na	644/0	0	616/6	0.8	na	na	na	na	648/0	0	616/6	1.0

LOAD 17/18	chr2:127932547 G>T	BIN1	na	na	na	na	485/0	0	655/6	0.8	na	na	na	na	487/0	0	659/6	0.9
Non-AD_Old 23/24	chr2:128078395 G>T	BIN1	na	na	na	na	439/0	0	534/6	0.9	448/0	0	538/6	1.1	na	na	na	na
Non-AD_Old 5/6	chr2:128123562 C>G	BIN1	na	na	na	na	1342/0	0	417/5	1.1	1399/0	0	422/5	1.2	na	na	na	na
LOAD 17/18	chr2:128130001 G>A	BIN1	na	na	na	na	765/0	0	800/9	1.5	766/0	0	802/10	1.2	na	na	na	na
EOAD 27/28	chr8:26755967 G>T	CLU/ PTK2B	na	na	na	na	785/0	0	488/5	0.9	na	na	na	na	786/0	0	487/5	1.0
Non-AD_Old 5/6	chr8:26778167 T>G	CLU/ PTK2B	na	na	na	na	234/0	0	178/5	2.5	266/0	0	186/6	3.1	na	na	na	na
LOAD 1/2	chr8:26790228 C>A	CLU/ PTK2B	na	na	na	na	na	na	na	na	402/0	0	294/5	1.7	401/0	0	293/5	1.7
Non-AD_Adult 29/30	chr8:26800386 G>T	CLU/ PTK2B	na	na	na	na	na	na	na	na	381/0	0	418/8	1.9	380/0	0	415/7	1.6
Non-AD_Adult 13/14	chr8:26809089 T>C	CLU/ PTK2B	na	na	na	na	na	na	na	na	168/0	0	135/5	3.5	166/0	0	131/5	3.6
EOAD 11/12	chr8:26809521 A>C	CLU/ PTK2B	na	na	na	na	1013/0	0	100/5	5.1	na	na	na	na	993/1	0	229/5	2.1
LOAD 17/18	chr8:26840966 T>G	CLU/ PTK2B	na	na	na	na	602/1	0	545/8	1.9	604/1	0	547/8	1.4	na	na	na	na
Non-AD_Adult 13/14	chr8:26841044 G>T	CLU/ PTK2B	na	na	na	na	416/0	0	567/6	0.8	na	na	na	na	418/0	0	591/6	1.0
LOAD 1/2	chr8:26930598 T>C	CLU/ PTK2B	673/0	0	630/5	0.80	981/0	0	1283/11	0.6	na	na	na	na	na	na	na	na
LOAD 3/4	chr8:26977698 T>C	CLU/ PTK2B	na	na	na	na	898/1	0	627/6	1.0	na	na	na	na	898/0	0	627/6	0.9
LOAD 1/2	chr8:27011648 A>G	CLU/ PTK2B	na	na	na	na	439/0	0	476/6	1.1	na	na	na	na	439/0	0	479/6	1.2
LOAD 1/2	chr8:27086347 T>A	CLU/ PTK2B	na	na	na	na	na	na	na	na	324/0	0	292/9	3.0	323/0	0	291/8	2.7
LOAD 1/2	chr8:27120664 G>T	CLU/ PTK2B	na	na	na	na	430/0	0	586/7	1.1	na	na	na	na	428/0	0	598/6	1.0
LOAD 1/2	chr8:27159092 G>T	CLU/ PTK2B	na	na	na	na	na	na	na	na	349/0	0	257/5	1.9	348/0	0	250/5	1.9
EOAD 25/26	chr8:27203803 T>C	CLU/ PTK2B	na	na	na	na	200/0	0	179/5	2.2	na	na	na	na	199/0	0	180/5	2.7
EOAD 25/26	chr8:27268113 G>T	CLU/ PTK2B	na	na	na	na	876/1	0	622/6	0.7	na	na	na	na	922/1	0	650/6	0.9
Non-AD_Old 21/22	chr8:27294615 G>T	CLU/ PTK2B	na	na	na	na	1129/0	0	436/5	0.9	na	na	na	na	1130/0	0	438/5	1.1
LOAD 19/20	chr8:27397739 A>G	CLU/ PTK2B	na	na	na	na	779/1	0	599/6	0.9	na	na	na	na	780/1	0	602/6	1.0
LOAD 17/18	chr8:27444641 G>C	CLU/ PTK2B	na	na	na	na	590/0	0	736/11	1.6	598/0	0	740/10	1.3	na	na	na	na

EOAD 27/28	chr8:27479442 G>T	CLU/ PTK2B	na	na	na	na	1301/1	0	594/6	0.8	na	na	na	na	1299/0	0	594/6	1.0
LOAD 17/18	chr8:27496959 G>T	CLU/ PTK2B	na	na	na	na	491/0	0	641/6	0.8	na	na	na	na	490/0	0	640/6	0.9
EOAD 9/10	chr8:27513913 G>T	CLU/ PTK2B	na	na	na	na	569/0	0	490/5	0.9	na	na	na	na	568/0	0	491/5	1.0
LOAD 17/18	chr11:85751855 G>A	PICALM	na	na	na	na	1501/0	0	1092/10	0.9	1501/0	0	1093/10	0.9	na	na	na	na
LOAD 17/18	chr11:85751856 G>C	PICALM	na	na	na	na	1509/0	0	1098/10	0.9	1509/0	0	1098/10	0.9	na	na	na	na
LOAD 1/2	chr11:85777460 G>T	PICALM	na	na	na	na	528/0	0	406/8	1.7	529/0	0	449/8	1.8	na	na	na	na
Non-AD_Old 5/6	chr11:85777870 G>T	PICALM	na	na	na	na	485/0	0	247/5	1.9	595/0	0	261/5	1.9	na	na	na	na
Non-AD_Old 5/6	chr11:85830157 T>C	PICALM	na	na	na	na	510/1	0	439/8	1.8	527/1	0	441/8	1.8	na	na	na	na
EOAD 11/12	chr11:120904973 G>T	SORL1	na	na	na	na	na	na	na	na	1035/4	0	194/5	2.5	1035/2	0	194/5	2.5
EOAD 25/26	chr11:120914367 G>T	SORL1	734/0	0	632/5	0.80	1269/1	0	1026/8	0.8	na	na	na	na	na	na	na	na
Non-AD_Old 5/6	chr11:120920473 T>G	SORL1	na	na	na	na	na	na	na	na	234/0	0	230/10	4.2	233/0	0	215/10	4.2
EOAD 11/12	chr11:120931495 G>T	SORL1	na	na	na	na	1042/0	0	296/7	1.8	na	na	na	na	1040/0	0	315/5	1.6
Non-AD_Old 21/22	chr11:121034807 G>T	SORL1	743/0	0	701/6	0.80	na	na	na	na	na	na	na	na	923/0	0	946/9	0.9
LOAD 17/18	chr11:121053051 G>A	SORL1	na	na	na	na	na	na	na	na	318/0	0	308/5	1.6	315/0	0	308/5	1.6
Non-AD_Old 5/6	chr11:121066946 C>T	SORL1	na	na	na	na	316/1	0	71/5	2.5	336/0	0	72/5	6.5	na	na	na	na
Non-AD_Old 21/22	chr11:121256750 G>T	SORL1	885/0	0	875/5	0.60	1008/0	0	1275/7	0.2	na	na	na	na	na	na	na	na
EOAD 25/26	chr11:121332780 G>T	SORL1	766/0	0	699/5	0.70	1532/1	0	1370/8	0.6	na	na	na	na	na	na	na	na
LOAD 19/20	chr11:121382085 G>T	SORL1	723/0	0	548/5	0.90	1073/0	0	959/9	0.9	na	na	na	na	na	na	na	na
Non-AD_Old 23/24	chr11:121415560 G>T	SORL1	950/0	0	906/5	0.50	1870/0	0	1723/8	0.4	na	na	na	na	na	na	na	na
EOAD 11/12	chr11:121424032 C>A	SORL1	na	na	na	na	1410/0	0	502/7	1.0	na	na	na	na	1405/1	0	508/7	1.4
EOAD 11/12	chr11:121436267 G>T	SORL1	724/0	0	525/5	0.90	1024/0	0	733/6	0.8	na	na	na	na	na	na	na	na
Non-AD_Adult 29/30	chr21:27290094 T>G	APP	na	na	na	na	na	na	na	na	338/0	0	339/6	1.7	338/0	0	337/6	1.7
EOAD 11/12	chr21:27316472 A>G	APP	na	na	na	na	na	na	na	na	1215/1	0	392/5	1.3	1216/0	0	392/5	1.3

EOAD 11/12	chr21:27350554 G>T	APP	na	na	na	na	na	na	na	na	1331/1	0	464/6	1.3	1317/0	0	462/6	1.3
Non-AD_Old 23/24	chr21:27360625 G>T	APP	na	na	na	na	800/0	0	476/5	0.7	na	na	na	na	804/0	0	477/5	1.0
Non-AD_Old 5/6	chr21:27366237 T>G	APP	na	na	na	na	290/1	0	637/17	2.5	na	na	na	na	305/1	0	615/15	2.3
EOAD 27/28	chr21:27382184 T>G	APP	na	na	na	na	228/1	0.01	96/6	5.7	245/2	0.01	105/6	5.1	na	na	na	na
EOAD 27/28	chr21:27419815 G>T	APP	na	na	na	na	829/0	0	621/6	0.8	na	na	na	na	831/0	0	623/6	1.0
LOAD 17/18	chr21:27423131 C>T	APP	na	na	na	na	1077/1	0	1115/14	1.5	1082/1	0	1119/13	1.1	na	na	na	na
Non-AD_Adult 13/14	chr21:27465796 G>T	APP	na	na	na	na	607/0	0	424/5	1.2	na	na	na	na	615/0	0	430/5	1.1

In total 71 SNVs were identified by two somatic mutation callers, whereas most of them by MuTect2, Strelka2 and Varscan2. LOAD (late-onset AD), EOAD (early-onset AD). Sample ID according to Table S1, chromosomal position is according to hg19. For the sequencing results the number of reads for reference (Ref) and alternative (Alt) allele is reported, as well as the alternative allele frequency (AAF) for the brain and the blood.

Table S6: The allele frequency of chr1:207461994C>T in the brain DNA of non-carriers

	Sequencing		ddPCR		
Brain sample	Allelic depth	Number of reads for T-allele	Fractional Abundance [95%CI] (a/(a+b)%)	Total DNA	Total Haploid copies
EOAD 10	1180	0	0	40	11940
EOAD 12	839	0	0	44	13163
EOAD 26	1455	0	0	53	15923
EOAD 28	924	0	0	207	62203
LOAD 2	1052	0	0	55	16768
LOAD 4	1183	0	0	124	37563
LOAD 18	1347	0	0	156	47403
Non-AD_Adult 14	549	0	0	47	13982
Non-AD_Adult 30	1483	0	0	92	27562
Non-AD_Adult 32	1527	0	0	80	24362
Non-AD_Old 6	563	0	0	92	27462
Non-AD_Old 22	745	0	0	179	53602
Non-AD_Old 24	1585	0	0	95	28683

The brain sample ID are given according to Table S1. For the sequencing data the allelic depth and number of reads for the T-allele are given as reported by mpileup obtained from Varscan2. For the ddPCR results, values are given as fractional abundance with 95% confidence intervals (CI) as well as total DNA and haploid copies tested. EOAD (early-onset AD), LOAD (late-onset AD).

Table S7: Transcription binding sites as predicted by JASPAR (upper panel) and PROMO (lower panel) for the sequence surrounding the wild-type (C) and mutant (T) allele of chr1:206461994

chr1:206,461,994-C | chr1:206,461,994-T

JASPAR

Model ID	Model name	predicted site sequence	Score	Relative score	Strand		Model ID	Model name	predicted site sequence	Score	Relative score	
							MA0058.2	MAX	AAGCACATTG	6.737	0.87	1
MA0442.1	SOX10	CAGTGT	6.812	0.91	-1							
							MA0084.1	SRY	GTAACCAAT	7.347	0.86	-1
MA0157.1	FOXO3	TGTAACCA	8.195	0.87	-1		MA0157.1	FOXO3	TGTAACCA	8.195	0.87	-1

PROMO

Factor	name	predicted site sequence	Dis similarity	RE equally	RE query		Factor	name	predicted site sequence	Dis similarity	RE equally	RE query
							[T00094]	NFI/CTF	CACATTGG	10.58	0.014	0.01
							[T00581]	C/EBPbeta	TTGG	1.64	0.32	0.37

For both softwares names and predicted site sequence is given. The variant is marked in red. For JASPAR the strand, score and relative score is given, for PROMO dissimilarity and RE scores are given.
